# Patterns and predictors of private and public health care utilization among residents of an informal settlement in Nairobi, Kenya: a cross-sectional study

**DOI:** 10.1186/s12889-021-10836-3

**Published:** 2021-05-03

**Authors:** Elvis O. A. Wambiya, Peter O. Otieno, Martin Kavao Mutua, Hermann Pythagore Pierre Donfouet, Shukri F. Mohamed

**Affiliations:** 1 African Population and Health Research Center, APHRC Campus, 2nd Floor, Manga Close, Off Kirawa Road, P.O. Box: 10787-00100, Nairobi, Kenya; 2 Department of Global Health and Population, Lown Scholars Program, Harvard T.H. Chan School of Public Health, Boston, MA USA

**Keywords:** Health care utilization, Informal settlement, Private facilities, Public facilities

## Abstract

**Background:**

Knowledge of health care utilization is important in low-and middle-income countries where inequalities in the burden of diseases and access to primary health care exist. Limited evidence exists on health seeking and utilization in the informal settlements in Kenya. This study assessed the patterns and predictors of private and public health care utilization in an urban informal settlement in Kenya.

**Methods:**

This study used data from the *Lown scholars* study conducted between June and July 2018. A total of 300 households were randomly selected and data collected from 364 household members who reported having sought care for an illness in the 12 months preceding the study. Data were collected on health-seeking behaviour and explanatory variables (predisposing, enabling, and need factors). Health care utilization patterns were described using proportions. Predictors of private or public health care use were identified using multinomial logistic regression with the reference group being other providers.

**Results:**

Majority of the participants used private (47%) and public facilities (33%) with 20% using other providers including local pharmacies/drug shops and traditional healers. In the model comparing public facilities vs other facilities, members who were satisfied with the quality of health care (vs not satisfied) were less likely to use public facilities (adjusted relative risk ratio (aRRR) 0.29; CI 0.11–0.76) while members who reported an acute infection (vs no acute infection) were more likely to use public facilities (aRRR 2.31; 95% CI 1.13–4.99) compared to other facilities. In the second model comparing private facilities to other facilities, having health insurance coverage (aRRR 2.95; 95% CI 1.53–5.69), satisfaction with cost of care (aRRR 2.08; CI 1.00–4.36), and having an acute infection (aRRR 2.97; 95% CI 1.50–5.86) were significantly associated with private facility use compared to other facilities.

**Conclusions:**

The majority of urban informal settlement dwellers seek care from private health facilities. As Kenya commits to achieving universal health coverage, interventions that improve health care access in informal and low-resource settlements are needed and should be modelled around enabling and need factors, particularly health care financing and quality of health care.

**Supplementary Information:**

The online version contains supplementary material available at 10.1186/s12889-021-10836-3.

## Background

Understanding patterns of health care utilization and knowledge of associated factors are important in improving health service delivery and ensuring equitable access to health services [1]. Health care utilization is widely used as an operational proxy for health care access [2]. Knowledge of health care utilization is particularly crucial in low-and middle-income countries (LMICs) where inequalities in the burden of disease and access to primary health care exist and may be attributable to the unrealised health gains observed in these settings [2, 3]. In fact, poor access to- and utilization of health care services are among the key reasons for the high morbidity and mortality rates in these regions [4].

Rapid urbanization in developing countries, especially in sub-Saharan Africa, and the corresponding increase in the urban population has given way to the burgeoning of informal settlements commonly referred to as slums [5]. Slums are associated with conditions that exacerbate poverty and high rates of disease attributable to overcrowding and poor sanitation [6–8]. As more evidence becomes available on the burden of disease in informal settlements, it is important to assess the patterns of health care utilization and influencing factors. Universal health coverage in LMICs has largely focused on the public health care sector as it has been argued that public health service provision is the best guarantee for equitable health care access and improved health outcomes for entire populations.

The Kenyan health system is mainly served by three categories of providers: public providers, private not-for-profit organisations – including mission hospitals and faith-based organizations -, and private for-profit organizations [9, 10]. The majority of the Kenyan population is served by public health care providers that are mostly operated by the government [10, 11]. However, private facilities are the majority of health service providers in urban informal settlements in Kenya and their population keeps increasing with the growing slum population. The private sector in Kenya operates about 43% of health centres, many of which are found in urban centres. There has been a growing increase in the use of unlicensed providers, drug shops and traditional healers in the slums, potentially delaying opportunities for optimal intervention [12, 13]. Kenya’s capital, Nairobi has over 200 informal and squatter settlements in which an estimated 60% of the urban population live [14].

Studies in sub-Saharan Africa have assessed factors influencing access to- and utilization of health care especially in rural and low-resourced populations [2, 3, 15–24]. In these settings, the main barriers to access of health care are cost of care, proximity to health facilities, perceptions on quality of care and disease status (both perceived and actual). Studies on health care seeking have been conducted in rural [25, 26] and urban settings [27–29] in Kenya. Factors found to influence health care seeking in these settings include socio-economic status, severity of illness, availability and acceptability of health services, quality of health care received and cost of care [25–30]. Studies conducted in urban slums have defined health seeking in terms of use or non-use of health facilities without distinction by the type of health facilities [27–29]. Furthermore, it is not clear how identified factors affect private and public health care utilization in these settings.

Despite there being considerable information on health care access and health care seeking behaviour in Kenya, the available evidence lacks distinction between factors influencing public and private health care utilization in urban informal settings which may impede the implementation of effective interventions and hinder efforts towards the achievement of health equity. This study sought to assess the patterns and predictors of private and public health care utilization among residents of an urban informal settlement in Nairobi, Kenya.

## Methods

### Study design and setting

Data for this study were obtained from the *Lown Scholars* study which aimed to investigate the feasibility of setting up a social health enterprise in the slum setting. This was a cross-sectional study conducted in Viwandani, an informal settlement in Nairobi, Kenya. Viwandani is adjacent to the Nairobi city’s industrial area. The area has predominately migrant young males working in the nearby industries. The *Lown Scholars* study was nested on the Nairobi Urban Health and Demographic Surveillance System (NUHDSS). Since 2003, the African Population and Health Research Center (APHRC) has been running the NUHDSS in two informal settlements (Korogocho and Viwandani). The NUHDSS captures routine information on births, deaths and migration from households twice a year. In 2012, 36,200 people were living in Viwandani [31]. The current population is approximately 52,698 people in about 22,739 households.

### Study population

The study sample included all household members from selected households who were reported to be ill in the 12 months preceding the study captured by the question: “Has any member of your household been ill in the last 12 months?” Household heads, their spouses or a credible adult (≥18 years old) provided information about household members who were ill, including themselves. Study participants were included in the study if they were adults (≥18 years old) and had resided in Viwandani for at least 12 months preceding the study.

### Sampling of households

The households were selected using simple random sampling from the NUHDSS households sampling frame which includes a listing of all households. The sample size for the original study was calculated using the formula for calculating sample size for cross-sectional studies (Cochran 1977); n= *z*^2^ × *p* × (1 − *p*)/*e*^2^ where z, p and *e* are: the standard normal deviation set at 95% confidence level (*z* = 1.96), the population proportion assumed to be willing to subscribe to the *Lown Scholars* social health enterprise (25%) and the margin error (e = 5%) respectively. By using a non-response rate of 4%, the final sample was 300. To select the 300 households, simple random sampling was performed in the NUHDSS database using a random number generator in MS Excel software. The first 300 households from the randomized list were selected as the sample for the study. All household members from the 300 households who reported illness in the 12 months preceding the study were included in the study bringing the total to 364 household members.

### Data collection

Data were collected electronically on a tablet using an interviewer-administered structured questionnaire that was developed by the *Lown Scholars* study research team. The questionnaire collected data on health facility utilization, sociodemographic variables including age, sex, marital status and educational status, possession of health insurance, socioeconomic status, employment status of the household head, accessibility (distance to health facility), satisfaction with health care, and perceived and physical health status of household members (Supplementary file 1). The study was approved by Amref Ethics and Scientific Review Committee (ESRC) and informed consent was obtained from all respondents prior to participation in the survey. The tool was pre-tested in a similar low-resource community prior to use for this study. The interviews lasted between 30 to 45 min. Data were collected between June and July 2018.

### Measurements

#### Outcome variable

Health care use was measured by the type of health facility that household members used in their last illness episode during the 12 months preceding the study. The options were grouped into three categories: public facility (public hospital/health centre) (2) private facility (private hospital/ private health centre or clinic/NGO mission hospital or health centre) and others (pharmacies/ chemists/ drug shops/traditional healers/herbalists).

#### Conceptual framework

We adapted Andersen’s conceptual framework for health care utilization to hypothesize factors influencing health care utilization (predictors). The framework posits access to- and utilization of health services to be a function of predisposing, enabling and need factors [32]. Predisposing factors are sociocultural characteristics that exist prior to the onset of illness that predict health care use upon illness [33]. They include demographic characteristics: age, sex, marital status, past illness etc.; social structure such as education, race, occupation, ethnicity and health attitudes and/or beliefs. Enabling factors are logistical conditions that enable one to obtain care including family resources such as income, health insurance coverage and community level factors including availability of health personnel and services [33]. Need factors are the most immediate cause of health service utilization – functional and health problems that generate the need for heath service use [33]. These represent the perceived or evaluated illness of an individual that prompt health service use. Examples include perceived health state and severity of illness.

#### Predictor variables

Predisposing factors included in our study included age of household member who were ill, their education level, sex, and marital status of the household head as well as the household size. We categorized age into five groups (Under 5 years, 5–17, 18–29, 30–44 and 45 and above); categories of marital status were married, divorced/separated/widowed and never married; and education level was grouped into primary school complete or less, secondary complete and university/college completed.

Enabling factors included possession of health insurance, socioeconomic status, employment status of the household head, accessibility (distance to health facility), satisfaction with health care service delivery and household expenditure on health per household member. Wealth quintiles were computed using NUHDSS data on household utilities (source of drinking water, type of toilet facility, cooking fuel used and lighting type at night), household characteristics (materials used to construct floor, wall and roof of dwelling) and household possessions (ownership of household items) for the households included in the study. Principal component analysis was used to first generate a wealth index from the household possessions variables which were later grouped into quintiles. The lowest wealth quintile (Q1) represents the poorest and the highest wealth quintile (Q5) represents the richest households.

Employment status included employed, casual worker, trader and unemployed. Satisfaction with health care services measured whether respondents were satisfied with waiting time, friendliness and respect of the provider, privacy of consultation and treatment received, quality of advice and information, procedure of treatment, cost of health services, and quality of services received at the primary care facility they visited for routine care. The variables were coded as 1-not satisfied at all, 2-slightly satisfied, 3-moderately satisfied, 4-very satisfied, 5-extremely satisfied. This variable was further recoded into two groups 1-satisfied (moderately satisfied, very satisfied, extremely satisfied) and 0-not satisfied (slightly satisfied, not satisfied at all).

We included both perceived and physical health status as need factors in the analysis. Perceived general health status of household members was measured in three categories: very good, moderate and not good, while physical health status included the medical condition that the household members sought care for. We categorized the health states into six broad categories including: acute infections (malaria, gastrointestinal conditions, typhoid, and respiratory tract infections), mild infections and others which included illnesses that were in low frequency in the sample, chronic infections (tuberculosis, HIV/STIs), chronic non-communicable diseases (NCDs) (diabetes, hypertension and chronic kidney disease), injuries or trauma and other uncommon conditions (hormonal conditions, autism, meningitis, eye and ear complications, skin conditions and dental conditions).

**Fig. 1:**
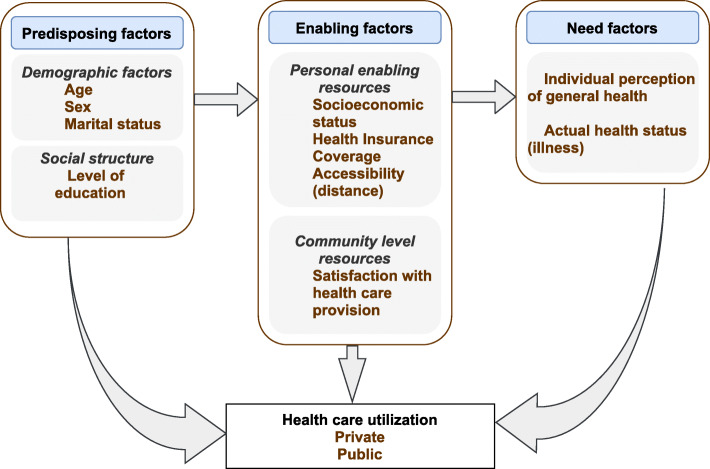
Adapted conceptual framework of factors affecting health care utilization

The adapted framework is shown in Fig. 1.

#### Data analysis

Descriptive statistics showing counts and proportions of predictor variables including age, wealth quintile, health insurance status, education status of household head, employment status of household head, satisfaction with health services and health condition prompting health care utilization are presented by health care utilization source (public, private and other facilities). Chi-square test of proportions was conducted to compare differences in proportions between explanatory variables by the outcome variable.

Bivariate analysis was conducted for predisposing, enabling and need factors to identify variables independently associated with health service use. Multinomial logistic regression was applied to identify factors associated with private or public health care use using ‘other facilities’ (pharmacies/ chemists/ drug shops/traditional healers/herbalists) as the reference group. In order to select variables for inclusion in the multivariable model, backward stepwise regression was conducted starting with all explanatory variables. This included stepwise elimination of least significant variables (Those with the highest *p* values) at every step until a parsimonious model is achieved. This was achieved when most variables had a *p* value< 0.05 [34]. The likelihood-ratio test (LRT) was used to assess the goodness-of-fit of the adjusted final reduced model against the initial full model containing all explanatory variables. The multinomial logistic regression model was adjusted for clustering at household level considering data was collected from all individuals who reported being ill and seeking care for the illness from each household. Adjusted relative risk ratios (aRRR) and 95% confidence intervals were reported for the predictors.

## Results

### Socio-demographic characteristics of study sample

Sociodemographic characteristics are presented in Table 1. In total, 364 individuals from the 300 households sought health care for an illness in the 12 months preceding the study. Approximately three quarters (73%) of the sample sought health care in facilities within Viwandani while the rest sought care outside the settlement. There were more females (60%) and approximately two thirds (61%) of the households had at least three members in the sample. The average age of household members was 22 years, inter-quartile range (IQR 6–35) and more than half were from households where the household heads were married. Approximately 21% of household heads were employed and 53% had at least secondary education. Household heads who had completed tertiary education accounted for only 7% of the sample.

**Table 1:** Sociodemographic characteristics of the study sample

	Frequency (n)	Percentage (%)
**Age**		
Under 5 years	75	20.6
5–17	80	22.0
18–29	85	23.4
30–44	86	23.6
45+	38	10.4
**Sex**		
Female	219	60.2
Male	145	39.8
**Marital status**		
Married	236	64.8
Divorced/separated/widowed	51	14.0
Never married	77	21.2
**Wealth quintile**		
Q1 (poorest)	61	16.8
Q2	62	17.0
Q3	81	22.3
Q4	85	23.4
Q5 (richest)	75	20.6
**Education status of household head**		
Primary complete or less	146	40.1
Secondary complete	193	53.0
College/University complete	25	6.9
**Employment status of household head**		
Employed	76	20.9
Casual worker	112	30.8
Trader	111	30.5
Unemployed	65	17.9
**Location of health facility visited**		
Within Viwandani	266	73.1
Outside Viwandani	98	26.9
**Total**	**364**	**100.0**

### Distribution of study sample by patterns of health care utilization

Table 2 shows a detailed distribution of the study sample by the type of health facility visited. Almost half (47%) of the participants sought care from private facilities while about 33 and 20% utilized public and other facilities respectively. Among respondents who sought care, 24% visited a public hospital while 9% visited a public health centre (Table 2). Most of those who sought care from private facilities visited a hospital (24%) or health centre (18%) while 3 and 2% visited an NGO mission hospital and health centre respectively (Table 2). Twenty percent of the sample sought care from local pharmacies or drug shops while 1% visited traditional healers.

**Table 2:** Detailed distribution of study sample by type of health care facility visited

		Frequency	Percentage
		**(n)**	**(%)**
**Public facilities**	Public hospital	87	23.9
	Public health centre	32	8.8
**Private facilities**	Private hospital	88	24.2
	Private health centre/clinic	64	17.6
	NGO Mission hospital	12	3.3
	NGO Mission health centre	7	1.9
**Other facilities**	Local pharmacy/chemist/ drug shop	71	19.5
	traditional healer/ herbalist	3	0.8
**Total**		**364.0**	**100.0**

Table 3 presents the distribution of the study participants by their health care utilization patterns. Overall, about 46% of the household members were covered by a health insurance scheme. A higher proportion of household members covered by health insurance used private facilities (58%) as compared to public (29%) and other facilities (14%). While more members from the richest households (63%) sought care from private facilities than those from poorest households (34%), public health facilities were used by a higher proportion of members from poorest households (44%) than the richest households (20%). About half of the household members sought care for acute infectious disease conditions, 27% for mild infections and 20% for other diseases. Private facilities were used by more than half (55%) of the household members who fell ill with acute infections, while public facilities were used by 30% of household members who had acute infections. For household members who had mild conditions, 39% sought care from private facilities while about a third sought care from public (30%) and other facilities (31%).

**Table 3:** Distribution of study participants by patterns of health care utilization

	Public facility n (%)	Private facility n (%)	Other facilities n (%)	Total N
***Predisposing factors***				
**Age**				
Under 5 years	23 (30.7)	37 (49.3)	15 (20.0)	75
5–17	26 (32.5)	40 (50.0)	14 (17.5)	80
18–29	24 (28.2)	46 (54.1)	15 (17.7)	85
30–44	33 (38.4)	35 (40.7)	18 (20.9)	86
45+	13 (34.2)	13 (34.2)	12 (31.6)	38
**Sex**				
Female	76 (34.7)	104 (47.5)	39 (17.8)	219
Male	43 (29.7)	67 (46.2)	35 (24.1)	145
Marital status**				
Married	63 (26.7)	124 (52.5)	49 (20.8)	236
Divorced/separated/widowed	20 (39.2)	21 (41.2)	10 (19.6)	51
Never married	36 (46.8)	26 (33.8)	15 (19.5)	77
***Enabling factors***				
Wealth quintile**				
Q1 (poorest)	27 (44.3)	21 (34.4)	13 (21.3)	61
Q2	23 (37.1)	21 (33.9)	18 (29.0)	62
Q3	23 (28.4)	41 (50.6)	17 (21.0)	81
Q4	31 (36.5)	41 (48.2)	13 (15.3)	85
Q5 (richest)	15 (20.0)	47 (62.7)	13 (17.3)	75
Health insurance status***				
Covered by insurance	48 (28.6)	97 (57.7)	23 (13.7)	168
Not covered by insurance	71 (36.2)	74 (37.8)	51 (26.0)	196
**Education status of household head**				
Primary complete or less	48 (32.9)	66 (45.2)	32 (21.9)	146
Secondary complete	67 (34.7)	87 (45.1)	39 (20.2)	193
College/University complete	4 (16.0)	18 (72.0)	3 (12.0)	25
**Employment status of household head**				
Employed	14 (18.4)	49 (64.5)	13 (17.1)	76
Casual worker	43 (38.4)	44 (39.3)	25 (22.3)	112
Trader	40 (36.0)	49 (44.1)	22 (19.8)	111
Unemployed	22 (33.9)	29 (44.6)	14 (21.5)	65
***Need factors***				
**Physical health state**				
Acute infections**	58 (30.2)	106 (55.2)	28 (14.6)	192
Mild infections	30 (30.0)	39 (39.0)	31 (31.0)	100
Chronic infections**	5 (83.3)	1 (16.7)	0	6
Chronic NCDs	6 (42.9)	7 (50.0)	1 (7.1)	14
Other diseases	20 (38.5)	18 (34.6)	14 (26.9)	52
**Total**	**119 (32.7)**	**171 (47.0)**	**74 (20.3)**	**364**

Notes: Other facilities include pharmacies/ drug shops and traditional healers; ** χ^2^ *p*-value < 0.05, *** χ^2^ *p*-value < 0.01; Row percentages are presented

### Predictors of health care utilization

The results of the adjusted multinomial logistic regression models showing the factors associated with utilization of health care facilities are presented in Table 4. The final model had good fit compared to the initial full model including all explanatory variables (LRT *p*-value< 0.01). Model 1 compares public facility healthcare utilization versus other facilities while model 2 compares private facility healthcare utilization versus other facilities. For both models, enabling and need factors were significant predictors of public and private health care utilization. None of the predisposing factors were significant. For model 1 (comparing public facility healthcare utilization versus other facilities), members who were satisfied with the quality of care in their primary health care facility (as compared those who were unsatisfied) were about 70% less likely to seek care from public health care facilities compared to other facilities (aRRR 0.29; CI 0.11–0.76) while those who reported having had an acute infection (compared to other illnesses) were twice as likely to seek care from public facilities (aRRR 2.31; 95% CI 1.13–4.99) compared to other facilities, For model 2 (comparing private facility use versus other facilities), household members who had health insurance coverage (as compared to those without insurance) were about three times more likely to seek private health care compared to other facilities (aRRR 2.95; 95% CI 1.53–5.69). Members who were satisfied with the cost of care were twice as likely to use public facilities (vs other facilities) compared to those who weren’t satisfied (aRRR 2.08; CI 1.00–4.36). Furthermore, respondents who reported having had an acute infection (compared to other illnesses) were about three times more likely to seek care from private facilities (aRRR 2.97; 95% CI 1.50–5.86) as compared to other facilities.

**Table 4:** Adjusted multinomial regression model of predictors of health care utilization

	Model 1: Public vs other facilities	Model 2: Private vs other facilities
	aRRR (CI)	aRRR (CI)
**Insurance coverage**		
No (Ref)	1.00	1.00
Yes	1.38 (0.69–2.75)	2.95 (1.53–5.69)^***^
**Satisfaction with health care**		
Cost of service		
No	1.00	1.00
Yes	2.08 (1.00–4.36)^**^	0.76 (0.39–1.50)
Quality of health care		
No	1.00	1.00
Yes	0.29 (0.11–0.76)^**^	0.57 (0.20–1.65)
**Physical health state**		
Acute infectious		
No	1.00	1.00
Yes	2.31 (1.13–4.99)^***^	2.97 (1.50–5.86)^***^
Other diseases		
No	1.00	1.00
Yes	2.31 (0.96–5.57)	1.48 (0.51–4.31)

Notes: Variables included are those in the final model after backward elimination; LRT *p*-value < 0.01 for reduced model vs full model; *aRRR* Adjusted relative risk ratio; *CI* 95% Confidence interval; *Ref* Reference category, ** *p*-value < 0.05, *** *p*-value < 0.01

## Discussion

This study sought to investigate patterns and predictors of private and public health care utilization in the context of an urban informal settlement in Kenya. Our findings indicate a higher use of private facilities in an informal slum settlement. The findings suggest that several factors including health insurance coverage, quality of care, cost of care and those who experienced acute infections are associated with private health care utilization. Public health care utilization on the other hand is influenced by cost of care and acute infections. These findings contribute to the evidence base from informal settlements on health care usage and will inform strategies that promote equitable access to health services in informal settlements now that universal health coverage is a top government priority in Kenya [35].

The high proportion (47%) of people seeking care from a private facility is concerning because it is among a disadvantaged population [36]. However, this finding supports the current evidence of increased private health care facility use in informal settlements in Kenya [30]. This has been caused in part by the rapid growth of urban informal settlements in Kenya accompanied by a paralleled mushrooming of private health facilities in these settings [37, 38]. Contrary to our findings, the latest national household health expenditure and utilization survey (KHHEUS) indicated a higher outpatient use of public health care facilities (44%) compared to private facilities (29%) [11]. The stark difference in our findings and the national picture are concerning since the government’s commitment towards universal health coverage (UHC) targets are focused on the public health sector. Studies have recommended the regulation of private health facilities in urban slum settlements, and promotion of public-private partnerships to improve quality health care access in these settings [30].

Empirical literature from LMICs supports our findings that health insurance coverage is a strong predictor of private health care utilization [3, 18, 24, 39]. Cost of health care is a significant barrier to access of health services in LMICs where payment for health care is mainly out-of-pocket (OOP) [2, 21, 24, 40]. As a result, health care use is on the basis of wealth instead of need depriving those of lower economic status access to health services, leading to high morbidity and mortality from preventable diseases ([41, 42]3). Cost of care remains a paramount issue in accessing health care in Kenya especially in low resource settings and is a major contributor to health care inequity [39, 43, 44].. Kenya’s commitment towards universal health coverage aims at cushioning citizens against OOP expenditure on health which pushes approximately one million Kenyans into poverty each year [45, 46]. Strategies employed to reduce out of pocket costs include the abolishment of user fees in public health care facilities in 2013 and the scale-up of National Health Insurance Fund (NHIF) coverage [47]. However, given that the majority of residents in informal settlements use private facilities there is need to have specific policies and interventions that will mitigate catastrophic expenditure. Private-public partnerships and the promotion of social or community health enterprises are recommended as prospective interventions to promote and balance access and utilization of private and public health care in informal settlements [48, 49].

Patient satisfaction is an important indicator for measuring quality of health care and has been widely used to measure health system performance in high and low income settings [17, 50, 51]. In the current study, satisfaction with quality of care in the respondents’ primary health care facility was associated with lower utilization of public health care which could point towards dissatisfaction with the quality of public health care among study respondents. This finding is confirmed by a study conducted in public facilities in Kenyan slums which found that there was higher dissatisfaction with services provided such as lack of drugs, long waiting times and bad attitudes from the staff [52]. Satisfaction with cost of care at the facility was also associated with higher use of public facilities. Evidence from LMICs has found user fees to be a barrier to health care utilization [21]. In the Kenyan context, reduction of user fees at public facilities revealed increased health care utilization which in turn put pressure on the public health care system [53].

Our findings that the majority of the study population in Viwandani fell ill with acute and mild infectious diseases including gastrointestinal infections, typhoid, malaria and respiratory diseases support available evidence. Urban informal settlements in Kenya are characterized by overcrowding, poor sanitation and hygiene, and limited access to clean water which makes this population vulnerable to infectious and vector-borne diseases [31, 36, 54]. Our findings of higher use of public and private facilities for acute infectious conditions are similar to findings from studies conducted in similar settings [13, 55–57]. Non-licensed and licensed private providers have been reported to be the major source of care for common infectious disease-related illnesses in informal settlements mainly due to their availability [13, 27, 28]. Population-based surveillance in Kenya also confirms infectious and respiratory illnesses as a major cause of morbidity and mortality in both children and adults, with many deaths occurring at home [55, 56].

The main limitation of this study is the inclusion of only one informal settlement thus limiting the extent to which results for this study are generalizable to informal settlements. Another key limitation is the cross-sectional nature of the study that limits casual association. Data on health care utilisation was self-reported hence this could have introduced some margin of error. The use of a proxy in some instances may have introduced some errors thus leading to either overestimation or underestimation. Therefore, the study findings should be interpreted with the above limitations in mind. Nevertheless, valuable information on the patterns and predictors of private and public health care utilization was obtained and will make a significant contribution to the body of knowledge in this area while informing the design of interventions in similar urban informal settlements in order to improve access to public and private health care facilities in such settings.

## Conclusions

The current study has important public health implications. Study findings show that a large proportion of individuals with low incomes living in informal settlements are accessing health care from private facilities rather than in public facilities. For Kenya to achieve its top priority - universal health coverage, interventions that promote equitable access to health services in urban informal settlements in Kenya where multiple health inequalities exist are needed. These interventions should focus on improving health insurance coverage, patient satisfaction in public facilities and the physical state of the patients.

## Supplementary Information

**Additional file 1: Supplementary file 1.** APHRC English questionnaire Lown Project

## Data Availability

The datasets generated and/or analysed during the current study are not publicly available due to grant agreements on data use but are available from the corresponding author on reasonable request.

## Abbreviations

NUHDSS: Nairobi urban health and demographic surveillance system
LMICs: Low- and middle-income countries
APHRC: African population and health research center
PCA: Principal component analysis
HIV/STIs: Human immunodeficiency virus/ sexually transmitted infections
NCDs: Non-communicable diseases
OR: Odds ratio
aRRR: Adjusted relative risk ratio
CI: Confidence interval

## References

[1] Jacobs B, Bigdeli M, Annear PL, Van Damme W (2011). Addressing access barriers to health services: an analytical framework for selecting appropriate interventions in low-income Asian countries. Health Policy Plan.

[2] O'Donnell O (2007). Access to health care in developing countries: breaking down demand side barriers. Cadernos Saude Publica.

[3] World Health Organization (2010). Health systems financing: the path to universal coverage: World Health Organization Geneva.

[4] Kruk ME, Gage AD, Joseph NT, Danaei G, García-Saisó S, Salomon JA (2018). Mortality due to low-quality health systems in the universal health coverage era: a systematic analysis of amenable deaths in 137 countries. Lancet.

[5] Phillips A (2014). African urbanization: slum growth and the rise of the Fringe City. Harv Int Rev.

[6] Ezeh A, Oyebode O, Satterthwaite D, Chen Y-F, Ndugwa R, Sartori J (2017). The history, geography, and sociology of slums and the health problems of people who live in slums. Lancet.

[7] Oppong JR, Mayer J, Oren E (2015). The global health threat of African urban slums: the example of urban tuberculosis. Afr Geogr Rev.

[8] Riley LW, Ko AI, Unger A, Reis MG (2007). Slum health: diseases of neglected populations. BMC Int Health Hum Rights.

[9] Mugo, P., Onsomu, E., Munga, B., Nafula, N., Mbithi, J., & Owino, E. (2018). An assessment of healthcare delivery in Kenya under the devolved system.

[10] Netherlands Enterprise Agency. (2016). Kenyan Healthcare Sector Opportunities for the Dutch Life Sciences & Health Sector.

[11] Ministry of Health, G. o. K. (2014). 2013 Kenya Household Health Expenditure and Utilisation Survey: Government of Kenya Nairobi.

[12] Amuyunzu-Nyamongo M, Nyamongo IK (2006). Health seeking behaviour of mothers of under-five-year-old children in the slum communities of Nairobi, Kenya. Anthropol Med.

[13] Breiman RF, Olack B, Shultz A, Roder S, Kimani K, Feikin DR, Burke H (2011). Healthcare-use for major infectious disease syndromes in an informal settlement in Nairobi, Kenya. J Health Popul Nutr.

[14] African Population and Health Research Center (APHRC) (2014). Population and health dynamics in Nairobi's informal settlements: report of the Nairobi cross-sectional slums survey (NCSS) 2012.

[15] Adane M, Mengistie B, Mulat W, Kloos H, Medhin G (2017). Utilization of health facilities and predictors of health-seeking behavior for under-five children with acute diarrhea in slums of Addis Ababa, Ethiopia: a community-based cross-sectional study. J Health Popul Nutr.

[16] Ameh S, Gómez-Olivé FX, Kahn K, Tollman SM, Klipstein-Grobusch K (2014). Predictors of health care use by adults 50 years and over in a rural south African setting. Glob Health Action.

[17] Awoke MA, Negin J, Moller J, Farell P, Yawson AE, Biritwum RB, Kowal P (2017). Predictors of public and private healthcare utilization and associated health system responsiveness among older adults in Ghana. Glob Health Action.

[18] Gyasi RM, Phillips DR, Buor D. The role of a health protection scheme in health services utilization among community-dwelling older persons in Ghana. J Gerontol B. 2020;75(3):661–73. 10.1093/geronb/gby082 29982726

[19] Harris, B., Goudge, J., Ataguba, J., McIntyre, D., Nxumalo, N., Jikwana, S., & Chersich, M. (2011). Inequities in access to health care in South Africa (Vol. 32 Suppl 1). 10.1057/jphp.2011.35 21730985

[20] Kiwanuka SN, Ekirapa EK, Peterson S, Okui O, Rahman MH, Peters D, Pariyo GW (2008). Access to and utilisation of health services for the poor in Uganda: a systematic review of available evidence. Trans R Soc Trop Med Hyg.

[21] Lagarde M, Palmer N (2008). The impact of user fees on health service utilization in low-and middle-income countries: how strong is the evidence?. Bull World Health Organ.

[22] Musoke D, Boynton P, Butler C, Musoke MB (2014). Health seeking behaviour and challenges in utilising health facilities in Wakiso district, Uganda. Afr Health Sci.

[23] Oladipo JA (2014). Utilization of health care services in rural and urban areas: a determinant factor in planning and managing health care delivery systems. Afr Health Sci.

[24] Saksena P, Xu K, Elovainio R, Perrot J (2010). Health services utilization and out-of-pocket expenditure at public and private facilities in low-income countries. World Health Rep.

[25] Burton DC, Flannery B, Onyango B, Larson C, Alaii J, Zhang X (2011). Healthcare-seeking behaviour for common infectious disease-related illnesses in rural Kenya: a community-based house-to-house survey. J Health Popul Nutr.

[26] Ngugi AK, Agoi F, Mahoney MR, Lakhani A, Mang'ong'o D, Nderitu E (2017). Utilization of health services in a resource-limited rural area in Kenya: prevalence and associated household-level factors. PLoS One.

[27] Taffa N, Chepngeno G, Amuyunzu-Nyamongo M (2005). Child morbidity and healthcare utilization in the slums of Nairobi, Kenya. J Trop Pediatr.

[28] Taffa N, Chepngeno G, Amuyunzu-Nyamongo M (2005). Child morbidity and healthcare utilization in the slums of Nairobi, Kenya. J Trop Pediatr.

[29] Wairiuko J, Cheboi S, Ochieng G, Oyore J (2017). Access to healthcare Services in Informal Settlement: perspective of the elderly in Kibera slum Nairobi-Kenya. Ann Med Health Sci Res.

[30] Mukiira C, Fotso JC (2011). Perceived quality of and access to care among poor urban women in Kenya and their utilization of delivery care: harnessing the potential of private clinics?. Health Policy Plan.

[31] Beguy D, Elung'ata P, Mberu B, Oduor C, Wamukoya M, Nganyi B, Ezeh A (2015). Health & demographic surveillance system profile: the Nairobi urban health and demographic surveillance system (NUHDSS). Int J Epidemiol.

[32] Andersen RM (1995). Revisiting the behavioral model and access to medical care: does it matter?. J Health Soc Behav.

[33] Andersen RM, Newman JF. Societal and individual determinants of medical care utilization in the United States. Milbank Q. 2005;83(4). 10.1111/j.1468-0009.2005.00428.x. 4198894

[34] Chowdhury MZI, Turin TC (2020). Variable selection strategies and its importance in clinical prediction modelling. Fam Med Community Health.

[35] Ministry of health. (2018). Domestic resource mobilization for health: National Health Financing Dialogue for implementation of the health sector domestic financing sustainability plan: unlocking investors’ potential in the delivery of UHC in Kenya. Kenya.

[36] APHRC. (2014). Population and health dynamics in Nairobi’s informal settlements: report of the Nairobi cross-sectional slums survey (NCSS) 2012. Nairobi.

[37] Bazant ES, Koenig MA, Fotso JC, Mills S (2009). Women's use of private and government health facilities for childbirth in Nairobi's. Informal settlements. Stud Fam Plan.

[38] Owino, G. E. (2015). “Preferences and utilization of health care services among slum residents in Kenya: a case of Mathare Valley.

[39] Chuma J, Okungu V (2011). Viewing the Kenyan health system through an equity lens: implications for universal coverage. Int J Equity Health.

[40] Rutebemberwa E, Pariyo G, Peterson S, Tomson G, Kallander K (2009). Utilization of public or private health care providers by febrile children after user fee removal in Uganda. Malar J.

[41] Bonfrer I, van de Poel E, Grimm M, Van Doorslaer E (2013). Does the distribution of healthcare utilization match needs in Africa?. Health Policy Plan.

[42] Tey N-P, Lai, S.-l. (2013). Correlates of and barriers to the utilization of health services for delivery in South Asia and sub-Saharan Africa. Sci World J.

[43] Turin DR. Health care utilization in the Kenyan health system: challenges and opportunities. Inquiries J Stud Pulse. 2010;2(9):1. http://www.inquiriesjournal.com/a?id=284.

[44] Umeh CA (2018). Challenges toward achieving universal health coverage in Ghana, Kenya, Nigeria, and Tanzania. Int J Health Plann Manag.

[45] Barasa EW, Maina T, Ravishankar N (2017). Assessing the impoverishing effects, and factors associated with the incidence of catastrophic health care payments in Kenya. Int J Equity Health.

[46] Chuma J, Maina T (2012). Catastrophic health care spending and impoverishment in Kenya. BMC Health Serv Res.

[47] World Bank Group. (2014). Laying the Foundation for a Robust Health Care System in Kenya: Kenya Public Expenditure Review: The World Bank.

[48] Bakibinga P, Ettarh R, Ziraba AK, Kyobutungi C, Kamande E, Ngomi N, Osindo J (2014). The effect of enhanced public–private partnerships on maternal, newborn and child health services and outcomes in Nairobi–Kenya: the PAMANECH quasi-experimental research protocol. BMJ Open.

[49] Marek T, O’Farrell C, Yamamoto C, Zable I (2005). Trends and opportunities in public-private partnerships to improve health service delivery in Africa.

[50] Masango Makgobela AT, Ndimande JV, Ogunbanjo G, Bongongo T, Nyalunga SN. Households’ satisfaction with the healthcare services rendered by a ward-based outreach team in Tshwane district, Pretoria, South Africa. S Afr Fam Pract. 2018:1–4. 10.1080/20786190.2018.1524552.

[51] Valentine NB, Bonsel GJ, Murray CJ (2007). Measuring quality of health care from the user's perspective in 41 countries: psychometric properties of WHO's questions on health systems responsiveness. Qual Life Res.

[52] Wambua JM, Mbayaki R, Munyao PM, Kabue MM, Mulindi R, Change PM (2015). Client satisfaction determinants in four Kenyan slums. Int J Health Care Qual Assur.

[53] Okech TC, Lelegwe SL (2016). Analysis of universal health coverage and equity on health care in Kenya. Glob J Health Sci.

[54] Gulis G, Mulumba JA, Juma O, Kakosova B (2004). Health status of people of slums in Nairobi, Kenya. Environ Res.

[55] Breiman RF, Cosmas L, Njenga MK, Williamson J, Mott JA, Katz MA (2015). Severe acute respiratory infection in children in a densely populated urban slum in Kenya, 2007–2011. BMC Infect Dis.

[56] Feikin DR, Olack B, Bigogo GM, Audi A, Cosmas L, Aura B, Burke H, Njenga MK, Williamson J, Breiman RF (2011). The burden of common infectious disease syndromes at the clinic and household level from population-based surveillance in rural and urban Kenya. PLoS One.

[57] Mohamed AH, Dalal W, Nyoka R, Burke H, Ahmed J, Auko E, Shihaji W, Ndege I, Breiman RF, Eidex RB (2014). Health care utilization for acute illnesses in an urban setting with a refugee population in Nairobi, Kenya: a cross-sectional survey. BMC Health Serv Res.

