# Supplementary material for: Patterns and predictors of private and public health care utilization among residents of an informal settlement in Nairobi, Kenya: a cross-sectional study
Source: BMC Public Health. 2021 May 3;21:850. doi: 10.1186/s12889-021-10836-3 (PMC8091493; doi:10.1186/s12889-021-10836-3)
Supplement: Supplementary file 1 — Additional file 1: Supplementary file 1. APHRC English questionnaire Lown Project [file 12889_2021_10836_MOESM1_ESM.pdf]

**African Population and Health Research Center (APHRC) English questionnaire Lown Project**

# Assessing Gaps in Healthcare and Determining the Feasibility for the setup of a Social Enterprise- Viwandani Lown Community Health Center, Kenya

[THE PERSON COMPLETING THIS HOUSEHOLD QUESTIONNAIRE SHOULD BE THE HEAD OF HOUSEHOLD IF AVAILABLE. WHERE UNAVAILABLE, ANY SPOUSE OF THE HEAD OF HOUSEHOLD SHOULD BE SELECTED TO COMPLETE THE HOUSEHOLD QUESTIONNAIRE. IN THE CASE THAT NEITHER THE HEAD OF HOUSEHOLD NOR ANY SPOUSE IS AVAILABLE, PLEASE COMPLETE THE HOUSEHOLD QUESTIONNAIRE WITH ANY OTHER AVAILABLE CREDIBLE ADULT AGED 18+)

## 1.0 BACKGROUND

1.1. START TIME

1.2. FIELD WORKER'S CODE

1.3. DATE OF INTERVIEW

1.4. HOUSEHOLD HEAD NAME……………………………………….

1.5. HOUSEHOLD ID

1.6 GPS COORDINATES

| | | | | | | |
|---|---|---|---|---|---|---|
| S01 | | | | | | |
| E036 | | | | | | |

## INTRODUCTION AND CONSENT

Hello, my name is………………………and I am working with the African Population and Health Research Center (APHRC). We are conducting a research study to assess the gaps in healthcare in this community. Your household was selected for the survey. Your opinion is important because it will enable us determine the feasibility of setting up a social enterprise. Your participation in this research is completely voluntary. You may also refuse to answer any question which you do not want to answer, and no harm will occur to you or anyone in your family regardless of your participation decision. 'The information that you provide will be completely confidential. Your responses will be combined with the answers of other respondents involved in the study and reported in such a way that it will not identify you. The interview will take about 45 minutes.

1.7. Do you accept to participate in the study? **[1=YES; 2=NO; IF YES SKIP TO 1.9]**

1.8. IF THE RESPONDENT DOES NOT ACCEPT TO BE INTERVIEWED: Why don't you want to participate in this interview?

**1=Too busy/Do not have time; 2= Tired of Research; 3= Research not beneficial; 4= Not interested; 6=Other (specify) ______________________________**

Thank you for your time.

**[IF NO IN 1.7 COMPLETE 1.1-1.6 AND SKIP TO 12.0]**

1.9. IF THE RESPONDENT ACCEPTS TO BE INTERVIEWED:
Thank you for agreeing to participate in this study.

**RESPONDENTS' PARTICULARS AND OTHER INTERVIEW DETAILS**

1.10. FW: IS RESPONDENT THE REFERENCE PERSON NAMED IN **1.4**? **[IF 1, SKIP TO 1.15]** ☐
**1=YES; 2=NO**

1.11 What is your name? ........................................................................................................

1.12. FW: DOES RESPONDENT LIVE IN THIS HOUSEHOLD? **[IF 2, SKIP TO 12.0]** ☐
1=YES; 2=NO
FW: SELECT RESPONDENT'S ID IN HOUSEHOLD ☐☐☐☐☐☐☐

1.13 How are you related to (NAME OF INDIVIDUAL IN Q1.4)? ........................................

**OFFICE/FIELD CHECKERS' DETAILS**

1.15 Field Supervisor/Team Leaders' Code ☐☐☐☐

| **SECTION 1: SOCIODEMOGRAPHIC INFORMATION** | | |
|---|---|---|
| **I will start by asking you some questions regarding the demographic information of your household. Please answer to the best of your knowledge.** | | |
| **Q101** | Sex of the respondent | 1. Male<br>2. Female |
| **Q102** | What is your date of birth? | ☐☐ ☐☐ ☐☐<br>Day month year |
| **Q103** | How old are you? | ⌊_⌊_⌋ years |

| | | |
|---|---|---|
| **Q104** | Have you attended school? | 1. Yes<br>2. No<br>**IF NO SKIP TO Q106** |
| **Q105** | What is your highest level of education? | 1.No formal schooling<br>2. Primary incomplete.<br>3. Primary complete<br>4.Secondary incomplete<br>5.Secondary complete<br>6.College/University incomplete<br>7.College/University complete |
| **Q106** | What is your marital status? | 1.Married or living together;<br>2.Divorced / separated;<br>3.Widowed; |

| | | |
|---|---|---|
| | | 4.Never-married and never lived together<br>5.others______ (SPECIFY) |
| **Q107** | What is your religion | 1.Roman Catholic<br>2.Protestant/Other<br>3.Other Christians<br>4. Muslim<br>5.No religion<br>5.Other ______ (SPECIFY) |
| **Q108** | What is your ethnic group / tribe? | 1.Embu<br>2.Kalenjin<br>3.Kamba<br>4.Kikuyu<br>5.Kisii<br>6.Luhya<br>7.Luo<br>8. Maasai<br>9. Meru<br>10. Mijikenda/Swahili<br>11. Somali<br>12. Others________ SPECIFY |
| **Q109** | How do you contribute to the household living?<br><br>***Tick all that apply.*** | 1. Employed worker<br>2. Casual worker<br>3. Trader<br>4. Housewife<br>6.Other_______ (SPECIFY) |
| **Q110** | What is your main source of income? | 1.Employed worker<br>2.Casual worker<br>3.Trader<br>4.Housewife<br>6.Other______ (SPECIFY) |
| **Q111** | Do you read a newspaper or a magazine at least once a week, less than once a week or not at all | 1. At least once a weak<br>2. Less than once a week<br>3. Not at all<br>99. Don't Know/Not sure |
| **Q112** | Do you own a mobile phone | 1. Yes<br>2. No |

| | | |
|---|---|---|
| **Q113** | Do you use your mobile phone for financial transactions | 1. Yes<br>2. No |
| **Q114** | Do you have an account in a bank or a financial institution that you yourself use | 1. Yes<br>2. No |
| **Q115** | Have you ever used internet | 1. Yes<br>2. No<br>**IF NO SKIP TO Q117** |
| **Q116** | In the last 12 months how often did use the internet | 1. Almost everyday<br>2. At least once a weak<br>3. Less than once a week<br>4. Not at all<br>99. Don't Know |
| **Q117** | How many people, including yourself, live in your household?<br><br>**FW: EXCLUDE ALL VISITORS**) **INCLUDE ALL CHILDREN IN BOARDING SCHOOL** | Number of people<br>\|__\|__\| |
| **Q118** | How many children, live in your household?<br><br>**FW: INCLUDE ALL PERSONS AGED** | Number of children<br>\|__\|__\| |

| | | |
|---|---|---|
| | **BELOW 18 YEARS** | |
| **Q119** | Of the children living in your household, how many are aged below 5 years | Number of children aged below 5 years<br>\|__\|__\| |
| **Section 2: Health status**<br>The next group of questions is about your health status. Please answer to the best of your knowledge. | | |
| **Q201** | Would you say that in general your health is? | 1. Excellent<br>2. Very good<br>3. Good<br>4. Fair<br>5. Poor |
| **Q202** | Thinking about your physical health, which includes physical illness and injury. For how many days during the past 30 days was your physical health not good? | \|__\|__\| Number of days |
| **Q203** | Now thinking about your mental health, which includes stress, depression, and problems with emotions, for how many days during the past 30 days was your mental health not good? | \|__\|__\| Number of days<br>. |

| | | |
|---|---|---|
| **Section 3; Behavioural Measurements**<br>***I am going to ask you a few more questions on tobacco use, alcohol use, diet, history of raised blood pressure and physical activity. Please answer to the best of your knowledge*** | | |
| Tobacco use<br>I will start by asking you some questions Tobacco use | | |
| Q301 | Have you ever smoked tobacco or used smokeless tobacco? | 1.Yes<br>2.No<br>**IF NO SKIP TO 304** |
| Q302 | Do you currently smoke any tobacco products, such as cigarettes, cigars or pipes? | 1.Yes<br>2.No |
| Q303 | Do you currently use tobacco products daily? | 1.Yes<br>2.No |
| Q304 | Have you **ever** consumed any alcohol such as beer, wine, spirits, fermented cider, changaa, busaa, or any other local brew? | 1.Yes<br>2.No |
| Q305 | How old were you when you first started consuming alcohol? | \|__\|__\| years |
| Q306 | In the last 12 months, how frequently have you had at least one alcoholic drink? | 1.Daily<br>2.Weekly<br>3.Monthly<br>4.Occasionally |
| **Diet**<br>The next questions ask about the fruits and vegetables that you usually eat. | | |

| | | |
|---|---|---|
| | | |
| Q307 | In a typical week, how many days do you **eat fruits** | ☐☐ If Zero days, go to Q309 |
| Q308 | How many **servings** of fruit do you eat on **one** of those days? | ☐☐ |
| Q309 | In a typical week, how many days do you **eat vegetables**? | ☐☐ If Zero days, go to Q311 |
| Q310 | How many **servings** of vegetables do you eat on one of those days? | ☐☐ |
| | **Physical Exercise.** The next questions ask about physical exercise. | |
| Q311 | During the past month, other than your regular job, did you participate in any physical activities or exercises such as running or walking for exercise? | 1.Yes<br>2.No |
| | **History of raised blood pressure** The next questions ask about history of raised blood pressure. | |
| Q312 | Have you ever had your blood pressure measured by a doctor or other health worker? | 1.Yes<br>2.No |
| Q313 | Have you ever been told by a doctor or other health worker that you have raised blood pressure or hypertension in the past 12 months? | 1.Yes<br>2.No |
| Q314 | In the past two weeks, have you taken any drugs (medication) for raised blood pressure prescribed by a doctor or other health worker? | 1.Yes<br>2.No |
| Q315 | Have you ever seen a traditional healer for raised blood pressure or hypertension? | 1.Yes<br>2.No |
| Q316 | Are you currently taking any herbal or traditional remedy for your raised blood pressure? | 1.Yes<br>2.No |

## SECTION 4; HEALTH CARE UTILIZATION

***I am going to ask you a few questions regarding the health care utilization in Viwandani. Please think about the last time you needed healthcare.***

| | | | | |
|---|---|---|---|---|
| Q401 | Has any member of your household (adult, child or yourself) been ill in the last 12 months? | 1.Yes<br>2.No<br>96.Don’t know<br><br>**IF NO SKIP TO Q410** | | |
| Q402 | If YES (Q401), how many people were ill? | Number of people ☐☐ | | |
| Q403 | If yes (Q401), how old was the person who was ill? | | **Age (years )** | **Age Group** |
| | | P1 | ☐☐ | |

| | | | | |
|---|---|---|---|---|
| | ***If age is not known, fill in the person's age group? IF < 5 yrs=1, 5-14 yrs=2, 15+ yrs=3*** | P2 | ☐☐ | |
| | | P3 | ☐☐ | |
| Q404 | Was treatment sought for the illness? | 1.Yes<br>2. No<br>96 Don't know<br>**IF NO SKIP TO Q409** | | |
| Q405 | Where was treatment sought outside home? | 1.Public hospital<br>2.Public health center<br>3.Private hospital<br>4.Private health center/clinic<br>5.NGO mission hospital<br>6.NGO mission health center<br>7.Pharmacy/Chemist<br>8.Traditional healer/Herbalist<br>99.Others……..(Specify) | | |
| Q406 | Is the facility or place located in Viwandani? | 1.Yes<br>2.No<br>96 .Don't know | | |
| Q407 | Why was treatment not sought?<br><br>[Check all that apply] | 1.Transport costs<br>2.Transport time<br>3.Cost at health facility<br>6.Long waiting time<br>7.Mistreatment by health providers<br>Other __________ (specify) | | |

| | | | | | |
|---|---|---|---|---|---|
| Q408 | What illness did the person have?<br><br>1.Diarrhea;<br>2.Malaria;<br>3.Cough;<br>4.Diabetes;<br>5.Vomiting;<br>6.Convulsion/ Seizures;<br>7.Difficult fast breathing;<br>8.Measles;<br>9. Injuries;<br>10. Others……(Specify) | **P1** | **P2** | **P3** | **P4** |
| Q409 | How many times has the person fallen ill in the past 12 months | P1 ☐☐ P3 ☐☐<br>P2 ☐☐ P4 ☐☐ | | | |
| Q410 | Where do you regularly go when you are sick or need health care for any member of your household? | 1.Public hospital<br>2.Public health center<br>3.Private hospital<br>4.Private health center/clinic<br>5.Charity, church or mission hospital<br>6.Charity, church or mission health center/clinic<br>7.Private doctors office<br>8.Pharmacy/Chemist<br>9.Traditional healer/Herbalist<br>99.Others……..(Specify) | | | |
| Q411 | Is the facility or place located in Viwandani? | 1.Yes<br>2.No<br>96.Don't know | | | |
| Q412 | How many times in the past 6 months have you | Doctor ☐☐<br>Nurse ☐☐<br>Midwife ☐☐<br>Traditional Birth Attendant ☐☐<br>Clinical officer ☐☐ | | | |

| | | |
|---|---|---|
| | sought care from the following healthcare providers? | Lab Technician ☐☐<br>Pharmacist/Drug Shop ☐☐<br>Community Health Worker ☐☐<br>Traditional Healer ☐☐<br>Others: specify____________ |
| Q413 | What is your primary means of transport to health facility?<br><br>(If transportation is evenly split between 2 or more means, then choose most expensive means.) | 1.Boda boda<br>3.Public bus<br>4.Taxi<br>5. Walking<br>6.Car or motorbike owned by you or household member<br>7.Car or motorbike owned by a friend, family or neighbour<br>99.Other ___________<br>(Specify) |
| Q414 | Does anyone accompany you on your visits to the health facility? | 1.Spouse<br>2.Brother/Sister<br>3.Other relative<br>4.Friend<br>5.No one<br>99.Other<br>___________<br>(Specify) |
| Q415 | How far away from your primary health facility do you live? | DISTANCE (KM)<br>☐☐ |
| Q416 | On a typical visit to the health facility how long does it usually take to travel from your house to the facility on one way trip? | Hours ☐☐ minutes ☐☐ |

| | | |
|---|---|---|
| Q417 | On a typical visit to the health facility. What is the average amount of time that you normally wait to see a medical staff? | 1. Less than 30 min.<br>2. 30 min. to 1 hour<br>3. 1 hour to 1 ½ hours<br>4 1 ½ to 2 hours<br>5. More than 2 hours |
| Q418 | Now thinking about the last time you visited a health facility, what health service(s) did you receive? | 1.Physical check-up<br>2.Immunizations<br>3.Family planning<br>4.Oral contraceptives<br>5. Prenatal/antenatal care<br>6.Post natal care<br>7.Dental<br>9. Voluntary Counselling and Treatment (VCT)<br>10. Physiotherapy<br>11.Other Services_________<br>(specify) |
| Q419 | The last time you visited a health facility, did the facility provide everything you needed to help you manage your health concerns? | 1.Yes<br>2 No<br>3 Don't know<br>**IF YES SKIP TO Q426** |
| Q420 | Which specific health service(s) was the facility unable to provide | 1 ☐<br>2 ☐<br>3 ☐<br>4 ☐ |
| Q421 | Were you referred to another | 1Yes<br>2.No<br>3 Don't know |

| | | |
|---|---|---|
| | hospital or health facility for services that were unavailable in the facility you visited? | |
| Q422 | Which facility were you referred to? | ☐ |
| Q423 | Which specific health service(s) were you referred for? | 1 ☐<br>2 ☐<br>3 ☐<br>4 ☐ |
| Q424 | Did you visit the facility you were referred to? | 1Yes<br>2.No<br>**IF YES SKIP TO Q426** |
| Q425 | What were the reasons for NOT visiting the referral facility? | 1.Transport costs<br>2.Transport time<br>3.Cost at health facility<br>4.Stock out of medications<br>5.Long waiting time<br>6.Mistreatment by health providers<br>99.Others _________ (Specify) |
| | **Satisfaction with health services**<br>***I am going to ask you a few questions regarding satisfaction with health services.*** | |
| Q426 | Thinking of your last healthcare visit, how satisfied were you with the following? | |

| | | | | | | |
|---|---|---|---|---|---|---|
| | | **1. Not satisfied at all,**<br>**2. Slightly satisfied,**<br>**3. Moderately satisfied,**<br>**4. Very satisfied,**<br>5. **Extremely satisfied** | | | | |
| | | 1 | 2 | 3 | 4 | 5 |
| a) | The length of waiting time | | | | | |
| b) | The friendliness and respect received from the health provider | | | | | |
| c) | The level of privacy of consultation and & treatment received | | | | | |
| d) | The quality of advice and information given by the healthcare provider | | | | | |
| f) | The procedure or treatment received | | | | | |
| g) | Cost of health care services | | | | | |
| h) | Quality of service | | | | | |
| Q427 | Do you currently face any barriers in accessing health care? | 1.Yes<br>2.No<br>**If No Skip to 501** | | | | |
| Q428 | What are the barriers you have faced in accessing health care in this community? [Check all that apply] | 1.Transport costs<br>2.Transport time<br>3. Cost at health facility<br>4.Stock out of medications<br>5.Long waiting time<br>6.Poor service quality | | | | |

| | | |
|---|---|---|
| | | 7.Mistreatment by health providers<br>99.Other<br>__________<br>(Specify) |

| | | |
|---|---|---|
| ***Section 5: Household Health Care Spending***<br>I would like to ask you more specific questions about how much your household and all its members spent in cash or in-kind on all health care and services that did not require an overnight stay. We want expenses in the last 3 months. If payment was in-kind, please estimate a monetary value. Please exclude costs to be reimbursed by insurance. | | |
| In the last 3 months, how much did your household spend on: | | |
| Q501 | Registration and consultation fees by doctors, nurses, or trained midwives that did not require an overnight stay?<br>*Amount in Kenya shillings* | ☐☐☐☐<br>96.Don't know |
| Q502 | Health-care by traditional or alternative healers?<br>*Amount in Kenya shillings* | ☐☐☐☐<br>96.Don't know |
| Q503 | Diagnostic and laboratory tests, such as x-rays or blood tests?<br>*Amount in Kenya shillings* | ☐☐☐☐<br>96.Don't know |
| Q504 | Medications or drugs (prescription, non-prescription, traditional, homeopathic…)?<br>*Amount in Kenya shillings* | ☐☐☐☐<br>96.Don't know |
| Q505 | Dentists or dental care?<br>*Amount in Kenya shillings* | ☐☐☐☐<br>96..Don't know |
| Q506 | Ambulance or other transport?<br>*Amount in Kenya shillings* | ☐☐☐☐<br>96.Don't know |
| Q507 | Out of pocket cost associated with overnight stays in a hospital or health facility? (Costs such as bed, labs, doctors etc.)<br>*Amount in Kenya shillings* | ☐☐☐☐<br>96.Don't know |
| Q508 | Any other health care products or services that were not included above? Please specify:<br>*Amount in Kenya shillings*<br><br>[PROMPT FOR FOOD COSTS IF PROVIDED TO HOUSEHOLD MEMBERS DURING HOSPITAL/CLINIC STAYS; PROMPT FOR HOME BASED CARE PROVIDED FOR HOUSEHOLD MEMBERS (ADULT OR CHILD) DUE TO LONG-TERM CONDITIONS OR GETTING OLD AND WEAK] | ☐☐☐☐<br>96.Don't know |

| Finally, I want you to think about how you paid for your health care expenditures over the last 12 months. This includes costs for all fees, services and goods, including overnight stays. | |
|---|---|
| Q509 | In the last 12 months, which of the following financial sources did your household use to pay for any and all health expenditures?<br>***Check all that apply***<br><br>Katika miezi 12 iliyopita, ni ipi njia ya mapato yafuatayo ambayo familia yako ilitumia kulipia matumizi yoyote ya afya?<br>***Angalia zote zinazotumika*** |
| 1. Current income of any household members (salaries, business, pensions, paid benefits…) ☐<br>2. Savings ☐<br>3. Payment or reimbursement from a health insurance plan (including community health schemes) ☐<br>4. Sold items (land, property, furniture, livestock etc.. ☐<br>5. Relatives ☐<br>6. Borrowed from financial institutions or agencies (microfinance schemes, bank ☐ | |

| 99. Others____________________ (Specify) | | |
|---|---|---|
| Q510 | Did you ever borrow any money in the last 12 months to pay for health expenditures? | 1. Yes<br>2. No |
| Q511 | If you borrowed any money in the last 12 months to pay for health expenditures, are you expected to pay this back? | 3. Yes<br>4. No |
| Q512 | Have you started paying back the loan? | 1. Yes<br>2. No |
| Q513 | What is the monthly repayment on the loan including interest?<br><br>Malipo ya kila mwezi kwa mkopo pamoja na riba ni ngapi?<br>[FOR INFORMAL PAYMENT, PLEASE TEASE OUT THE AVERAGE MONTHLY REPAYMENT, IF ANY] | ☐☐☐☐<br>96. Don't know |

| | SECTION 6; HEALTH INSURANCE STATUS<br>***I am going to ask you a few more questions regarding your current health insurance status and that of the members of your household. Please answer to the best of your knowledge.*** | | | | |
|---|---|---|---|---|---|
| | | | | | |
| | Q601A | Q601B | Q601C | Q601D | Q601E |
| | Are you or any member of your household covered by any kind of health insurance?<br>1. Yes<br>2. No<br>96. Not sure<br>[IF "NO" skip to Q701, IF "YES" continue with Q601B-E] | What type of health insurance is (NAME) covered by?<br>1.Mutual health organisation/ community-based health insurance;<br>2.Health insurance through employer;<br>3. Privately purchased health insurance;<br>99. Other, please specify.<br>***FW: IF More than one insurance plan. List all that apply.*** | What is the name of the health insurance that covers (NAME).<br><br>1.National Health Insurance Fund (NHIF)<br>2.Jubilee<br>3.Madison<br>4.Resolution<br>5.APA<br>6.Apollo<br>7. Pacis<br>8. UAP<br>9. Britam<br>10.Heritage<br>11. AAR<br>12Pan Africa Life<br>13. Mtiba<br>96. Don't Know<br>99.Other……(specify).<br><br>***FW: IF More than one insurance plan. List all that apply.*** | How much does your household pay for [NAME]'s health insurance per month?<br><br>*If yearly amount is mentioned, calculate the monthly amount* | What medical services are covered by (NAME) health insurance?<br><br>(***Select all that apply - Multiple choices allowed)***<br>1. In patient<br>2. Out patient<br>3. Both Inpatient and Outpatient<br>4. Maternity cover<br>5. Others (specify)<br>8. Don't Know |
| 01 | | | | | |
| 02 | | | | | |
| 03 | | | | | |
| 04 | | | | | |
| 05 | | | | | |
| | Total monthly expenditure on health insurance | | | | AMOUNT IN KENYA SHILLINGS<br>☐☐☐☐☐ |

| SECTION 7: COMMUNITY'S EXPECTATION FOR A PRIMARY HEALTHCARE & THEIR WILLINGNESS TO PAY FOR SERVICES. | | |
|---|---|---|
| *(Read the following to the participant)*<br>**Imagine that your community with support from the government and non-governmental organizations has decided to set up a health insurance scheme to improve the quality of health services of the health center nearest to your home by bringing in trained and qualified doctors, laboratory, and pharmacy services, improving the availability of medications and consumables, and improving the environment for waiting places and reducing the waiting time. But the community members will be asked to make regular contributions to supplement the costs of running the clinic. The healthcare services provided in this health center will be better than what you are currently receiving, would be available to you and your household members, and would be either or highly discounted at the clinic. The contribution would be similar to an insurance premium, with no refund for those who do not need to use the health services.**<br>**Under this plan, you would be asked to pay a certain amount of money, similar to an insurance premium, which can be paid monthly~~, quarterly or annually~~.**<br><br>When answering the following question, I would like you to think about your monthly income and that your decision will be used in whether or not to establish the insurance scheme described above. I will present to you a list of monthly premium and I would like you to indicate the interval where your monthly premium lies.<br><br>(Ask whether the participant has understood the subject matter and whether they have any questions). | | |
| Q701 | Would you be willing to participate in the above described healthcare model? | 1. Yes<br>2. No<br>96. Not sure<br>**IF NO SKIP TO Q705** |
| Q702 | Who would you be willing to pay for in the health insurance scheme? (Please choose the best option for you) | 1. Only myself<br>2. Myself and my children<br>3. Myself and my spouse.<br>4. Myself, my spouse and my children<br>5. All household members<br>6. Extended family<br>7. Others (Please specify)_______________ |
| Q703 | Before you give me a response to your maximum monthly contribution, I would like to remind you that we conducted this similar study somewhere and we realized there is a difference between the maximum amount that people are willing to pay during the survey and what they are capable of paying. Please keeping in mind what we have discussed, I would like that you respond truthfully. Please can you respond truthfully about your monthly contribution for the health insurance scheme? | 1. Yes<br>2. No |

<table>
<tr><td></td><td>Kabla unipe jibu juu ya kiwango cha juu cha mchango wako wa mwezi, ningependa kukukumbusha kuwa tulitekeleza shughuli kama hiii pahali pengine tukapata kuwa kuna tofauti kati ya kiwango cha juu ambayo wangependa kulipa na wanawezalipa. Tafadhali ukitafakari tunayozungumzia ningependa ujibu kwa kweli. Je, unaweza jibu kwa ukweli kuhusu malipo yako ya kila mwezi ya bima ya afya.</td><td></td></tr>
<tr><td colspan="3">Now, we would now like to know about the amount of money that you would be willing to pay per month for the health insurance scheme in order for yourself and your household members up to four children.</td></tr>
<tr><td>Q704</td><td>Instructions to interviewers: "Ask the respondent if he/she would definitely pay Ksh 50 per month for the insurance scheme. If yes, tick the first cell in column A, then ask if they would definitely pay Ksh 100. Keep going until the respondent says "NO".<br>Then ask if they are sure Ksh 1000 is too much for them. If YES, place a cross in the lowest cell of column B, and ask them if Ksh 600 is too much. Keep going up column B until they say that they are not sure if Ksh X is too much."</td><td>"Read out column A from lowest to highest: read out column B from highest to lowest"<br><table><tr><td>Amounts in KES per month</td><td>A: I would definitely pay per month (tick)</td><td>B: I would definitely NOT pay per month (cross)</td></tr><tr><td>50</td><td></td><td></td></tr><tr><td>100</td><td></td><td></td></tr><tr><td>160</td><td></td><td></td></tr><tr><td>200</td><td></td><td></td></tr><tr><td>250</td><td></td><td></td></tr><tr><td>300</td><td></td><td></td></tr><tr><td>600</td><td></td><td></td></tr><tr><td>1000</td><td></td><td></td></tr></table><br>a. Lower bound. Ksh__________<br><br>b. Upper bound Ksh___________</td></tr>
<tr><td>Q705</td><td>Why would you not be willing to pay for health insurance?</td><td>1.Will not use the public healthcare services if we have to pay<br>2. My household cannot afford to make a contribution<br>3. Only financially comfortable people should pay the contributions<br>4. Prefer other ways of making the contributions –<br>5.Other (Please specify)________________________<br>PROBE FURTHER FOR OTHER REASONS WHY THEY ARE NOT WILLING TO PAY FOR HEALTH INSURANCE</td></tr>
</table>

| | | |
|---|---|---|
| | What specific health service(s) would like to be offered by the proposed primary health care program? | ------------------------------------------------------------------------------------------------------------------------------------------------------------------------------------------------------------------------------------------- |

## SECTION 8: MEASUREMENTS

| 8.0 ANTHROPOMETRIC MEASUREMENTS<br>*Next, I am going to take your weight and height measurements.*<br>*Ifuatayo, nitachukua kipimo cha uzito na urefu wako.* | | |
|---|---|---|
| **Q801** | Standing height | \|__\|__\|__\|__\| mm |
| **Q802** | Weight | \|__\|__\|__\|.\|__\| kg |
| **Q803** | Waist circumference | \|__\|__\|__\|__\| mm |
| **Q804** | Hip circumference | \|__\|__\|__\|__\| mm |

| 9.0 BLOOD PRESSURE<br>*Next, I am going to take your blood pressure measurements.*<br>***Ifuatayo, nitakuchukua kipimo cha shinikizo la damu*** | | |
|---|---|---|
| **Q901** | Systolic 1 | \|__\|__\|__\| mmHg |
| **Q902** | Systolic 2 | \|__\|__\|__\| mmHg |
| **Q903** | Systolic 3 | \|__\|__\|__\| mmHg |
| **Q904** | Diastolic 1 | \|__\|__\|__\| mmHg |
| **Q905** | Diastolic 2 | \|__\|__\|__\| mmHg |
| **Q906** | Diastolic 3 | \|__\|__\|__\| mmHg |
| **Q907** | Time blood pressure taken in the first instance<br>***[Based on a 24 hour clock eg.15:30]*** | **\| h \| h \|:\| m \| m \|** |

| 10. PULSE | | |
|---|---|---|
| **Q1001** | Pulse 1 | \|__\|__\|__\| beats/min |
| **Q1002** | Pulse 2 | \|__\|__\|__\| beats/min |
| **Q1003** | Pulse 3 | \|__\|__\|__\| beats/min |
| **Q1004** | Person performing measurements | ____________________________ |

| | | |
|---|---|---|
| **Q1005** | Notes or comments related to measurements | |

| | | | | |
|---|---|---|---|---|
| | **11. MUAC MEASUREMENT & OEDEMA FOR CHILDREN AGE 0-5**<br>*Next, I am going to take MUAC measurement of a child/children aged below 5 years living in this household* | | | |
| | **CHECK Q 103 AND RECORD THE AGES AND SEX FOR ALL ELIGIBLE CHILDREN 0-5 YEARS** | | | |
| **Q1101** | | **CHILD 1**<br>AGE ⌊__⌊__⌋ | **CHILD 2**<br>AGE ⌊__⌊__⌋ | **CHILD 3**<br>AGE ⌊__⌊__⌋ |
| **Q1102** | | Sex:<br>1. Male<br>2. Female | Sex:<br>1. Male<br>2. Female | Sex:<br>1. Male<br>2. Female |
| Q1103 | Mid Upper Arm Circumference | In Centimeters (cm).<br>⌊__⌊__⌊__⌋ . ⌊__⌋ | In Centimeters (cm).<br>⌊__⌊__⌊__⌋ . ⌊__⌋ | In Centimeters (cm).<br>⌊__⌊__⌊__⌋ . ⌊__⌋ |
| Q1104 | Oedema<br>*(Check for oedema)* | 1.Yes<br>2.No | 1.Yes<br>2.No | 1.Yes<br>2.No |
| | **SECTION 12: END OF INTERVIEW** | | | |
| **Q1201** | | I would like to thank you for taking your time to participate in the interview. As I said at the beginning, this information will help us to assess the gaps in healthcare in this community and determine the feasibility for setting up of a social enterprise.<br>Do you have any question for me?<br>1=YES; 2= NO; **[IF NO 2 SKIP TO Q1203]** | | |
| Q1202 | | FW: RECORD QUESTIONS AND COMMENTS RAISED BY RESPONDENT | | |
| Q1203 | | FW: RECORD COMMENTS ABOUT THE INTERVIEW | | |
| Q1204 | | **END TIME (24 HRS)** | | |
